# Investigation of Underlying Association between Anthropometric and Cardiorespiratory Fitness Markers among Overweight and Obese Adolescents in Canada

**DOI:** 10.3390/ijerph21040408

**Published:** 2024-03-28

**Authors:** Mario Leone, Hung Tien Bui, Emilia Kalinova, Jean Lemoyne, Dominic Gagnon, Luc Léger, Georges Larivière, Maxime Allisse

**Affiliations:** 1 Département des Sciences de la Santé, Université du Québec à Chicoutimi, 555 Boulevard de l’Université, Saguenay, QC G7H 2B1, Canada; hung.tien.bui@usherbrooke.ca (H.T.B.); maxime.allisse@usherbrooke.ca (M.A.); 2 Faculté de Médecine, Université de Sherbrooke, Sherbrooke, QC J1H 5N4, Canada; 3 Département des Sciences de L’activité Physique, Université du Québec à Montréal, Montréal, QC H2X 1Y4, Canada; kalinova.emilia@uqam.ca; 4 Département des Sciences de L’activité Physique, Université du Québec à Trois-Rivières, Trois-Rivières, QC G8Z 4M3, Canada; jean.lemoyne@uqtr.ca; 5 Jonquière Medic, Saguenay, QC G7X 7W6, Canada; dominic.gagnon.med@ssss.gouv.qc.ca; 6 École de Kinésiologie et des Sciences de L’activité Physique, Université de Montréal, Montreal, QC H3C 3J7, Canada; luc.leger@umontreal.ca (L.L.); georgeslariviere111@sympatico.ca (G.L.); 7 Faculté des Sciences de L’activité Physique, Université de Sherbrooke, Sherbrooke, QC H2X 2R1, Canada

**Keywords:** body composition, maximal functional aerobic power, VO_2_peak, youth, cardiometabolic risk

## Abstract

Background: Adolescents who experience overweight or obesity commonly persist in these conditions into adulthood, thereby elevating their vulnerability to health issues. The focus of this study is on health risk markers such as body mass index (BMI), waist circumference (WC), waist-to-height ratio (WHtR), body surface area (BSA), and cardiorespiratory fitness (CRF). The objectives include updating normative values for BMI, WC, WHtR, and BSA in Canadian adolescents, establishing cardiometabolic risk zones, and developing a composite score considering both anthropometric and CRF markers. Methods: Involving 1864 adolescents, the study used the LMS method to generate percentile norms, stratified by age and sex. Cardiometabolic risk zones were established for each marker based on Z-scores, and a composite score was created. Results: An increase in WC of 5.8 and 7.4 cm for boys and girls, respectively, was observed since 1981. Forward multiple regression analyses were conducted to assess the robustness and validity of the proposed model. The results indicated that the model explained nearly 90% (R^2^ = 0.890) of the common variance between the composite score and the retained independent variables. Moreover, the model demonstrated a mean absolute error (MAE) of approximately 6 percentiles, confirming its high precision. Furthermore, these analyses yielded key thresholds for identifying adolescents at risk: the 70th percentile for high cardiometabolic risk and the 85th percentile for very-high risk. Conclusions: Individually, WC or WHtR seem to be better markers for evaluating cardiometabolic risk than BMI during adolescence. However, CRF showed comparable importance to anthropometric markers in determining cardiometabolic risk. The simultaneous inclusion of anthropometric and CRF markers provides a better picture of the global cardiometabolic risk in adolescents.

## 1. Introduction

While all aspects of adolescent development are crucial for overall well-being, some areas have become increasingly concerning in recent years. One major issue is the alarming rise in the prevalence of overweight and obesity among teenagers [1]. Numerous studies have highlighted that a significant proportion of adolescents struggling with overweight or obesity are likely to carry these conditions into adulthood [2,3,4,5]. This persistence significantly increases the probability of early onset morbidity and mortality, primarily due to cardiovascular and cardiometabolic disorders [4,5]. These conditions can manifest early in childhood and progress into co-occurring illnesses during adolescence and young adulthood [5]. Therefore, from both clinical and public health standpoints, it is essential to ensure access to accurate and up-to-date data to enable effective monitoring of these risk factors.

### 1.1. Body Composition Assessment

A myriad of techniques exist for assessing body composition, including dual-energy X-ray absorptiometry (DEXA), computed tomography (CT), magnetic resonance imaging (MRI), bioelectrical impedance (BI), and hydrostatic weighing [6,7,8,9]. While undoubtedly valuable, these methods often face limitations in accessibility for widespread clinical and population-based surveillance. These limitations primarily stem from factors like limited device availability, high costs, reliance on specialized personnel, and the potential invasiveness of certain techniques [4,10]. Fortunately, alternative methods provide enhanced accessibility, facilitating the surmounting of these obstacles to proactively mitigate the associated risks.

#### Body Mass Index

Despite its limitations, body mass index (BMI) remains the most prevalent method for tracking overweight and obesity in children and adolescents [3,11,12,13]. Its widespread adoption stems from its remarkable simplicity, requiring only two readily available measurements, namely body mass and body height. Moreover, age- and sex-specific percentile charts empower healthcare professionals to accurately identify individuals at highest risk [14]. Indeed, a substantial body of evidence has firmly established a compelling association between elevated BMI values and various health conditions, including type 2 diabetes, hypertension, coronary artery disease, dyslipidemia, and other metabolic disorders [4,15,16,17].

Regardless the numerous advantages associated with BMI monitoring, such as its simplicity and strong correlation with various health indicators, several authors have expressed concerns regarding its accuracy as a measure of overweight or obesity, particularly among young individuals [3,13,18,19]. The most common argument raised is that BMI serves as a general measure (total obesity) and fails to differentiate between adipose tissue and lean body mass, providing no information on body fat distribution. This argument is well founded, given that BMI relies primarily on body mass and body height, both of which undergo highly heterogeneous changes during adolescence. Therefore, it is imperative to incorporate additional reliable markers that can validate the trends assessed by BMI.

### 1.2. Other Anthropometric Markers

Beyond BMI, several non-invasive clinical measures have been developed to estimate the degree of overweight and obesity, including the assessment of subcutaneous skinfold thickness, conicity index, body shape index, and waist-to-hip ratio, among others [10,15,20,21,22]. While innovative, these techniques face hurdles limiting their widespread implementation. For instance, their application necessitates additional anthropometric measurements, hindering their feasibility in busy clinical settings. Moreover, some of these techniques lack validation for children and adolescents, further complicating their interpretation. Despite these challenges, there are alternative methods that hold promise in enhancing the evaluation of overweight or obesity while imposing minimal additional constraints.

#### 1.2.1. Waist Circumference (WC)

Given that BMI and WC capture distinct aspects of obesity, incorporating both measures would likely yield a more comprehensive understanding of the condition. The importance of measuring waist circumference (WC) has gained significant traction in recent years, extending from clinical settings to population-wide studies. This simple and fast method provides a quick means of evaluation that can be easily applied in a clinical context. Moreover, using WC alongside BMI refines the accuracy of adiposity profiling in young individuals. Several studies have demonstrated that WC surpasses BMI as a marker of cardiovascular and cardiometabolic risk [3,4,18,23]. This advantage stems from its ability to assess abdominal adiposity (central obesity), recognized as the most detrimental form. Since BMI and WC capture distinct aspects of obesity, combining both measures would likely yield a more complete understanding of the condition. As such, it may be prudent to incorporate both markers as a minimum requirement.

#### 1.2.2. Waist-to-Height Ratio

The waist circumference-to-height ratio (WHtR) offers a promising alternative measure for abdominal obesity in adolescents. This metric is calculated by dividing an individual’s waist circumference by their body height, providing a more nuanced assessment compared to solely measuring WC. Some studies have suggested that WHtR may surpass both BMI and WC in predicting cardiometabolic and cardiovascular risk in adolescents [24,25,26,27,28]. The rationale underlying this index is that for a given body height, there is an acceptable amount of fat stored on the upper body. The commonly endorsed and widely accepted value for WHtR in a healthy individual is 0.5 or less [26]. Despite its widespread acceptance in adults, the validity of this threshold in children and adolescents is under scrutiny, as it does not account for the natural variation of WHtR (i.e., changes in body height) with age and sex in growing individuals [29]. Regardless of this limitation, we believe that WHtR still holds promise as a tool to identify high-risk adolescents, even those with normal BMI, warranting further research and potentially age- and sex-specific cut-offs.

#### 1.2.3. Body Surface Area

Body surface area (BSA) is a measure of the total surface area of the human body. Given the substantial variability in body size across individuals, BSA has emerged as a standardization tool for various clinical assessments of biological function [30]. BSA is particularly useful in children and adolescents because it gives a more accurate assessment of morphological changes during growth. This is because BSA takes into account both body height and body mass, which are two important factors that affect body size. This distinctive attribute imparts an allometric dimension to BSA, an aspect often underrepresented or inadequately captured by other anthropometric markers. While BSA may not be as prevalent as BMI, it is acknowledged as a health indicator capable of gauging an individual’s health status [31,32,33,34]. Notably, certain studies go beyond the conventional use of BMI and assert that BSA serves as a more robust predictor of heart failure mortality and numerous other diseases [34]. Therefore, BSA is a valuable tool for clinicians and researchers who are interested in assessing body size and composition.

Although the intercorrelation between these four markers is relatively high (i.e., BMI, WC, BSA, and WHtR), each offers a different perspective on body composition. Thus, the concept of incorporating multiple markers is interesting. This multi-marker approach simultaneously captures both global and local aspects of obesity, leading to a richer understanding of an individual’s adipose profile. Furthermore, using multiple markers enhances the certainty of the assessment compared to relying solely on one indicator. Finally, these four markers can be easily calculated as they are all derived from the same three anthropometric variables: body mass, body height, and WC.

### 1.3. Cardiorespiratory Fitness

Beyond anthropometric markers, other variables are also closely associated with cardiovascular or cardiometabolic risks. Of these variables, one of the most extensively studied physiological factors is cardiorespiratory fitness (CRF), which refers to the ability of the cardiorespiratory system to supply oxygen to the muscles during sustained physical exertion. Several studies have demonstrated a clear association between low CRF and cardiometabolic risk among children and adolescents [2,35,36,37,38].

The significance of this marker highlighted by the fact that the American Heart Association (AHA) advocates for its routine monitoring, deeming it a “vital sign” due to its ability to predict mortality in adulthood comparable to conventional assessments of cardiometabolic and cardiovascular risk factors [36,39]. Typically expressed in ml·kg^−1^·min^−1^, CRF is also referred to as VO_2_max or VO_2_peak. While various methods exist to measure or estimate CRF, those requiring the analysis of expired gas are generally considered the “gold standard”. However, the limited availability of equipment, the complexity of the procedures, the extended duration of the evaluation process, and associated costs make this type of measurement relatively inaccessible. To overcome these limitations, several indirect procedures have been developed to obtain an accurate and reliable evaluation of CRF. These procedures include field tests, laboratory tests, and self-reported questionnaires.

For decades, the most widely used method to assess CRF has been the 20 m shuttle run test [40]. Every year, millions of young people worldwide are assessed using this procedure, making VO_2_peak values available for this population [41,42]. This procedure is particularly attractive for CRF assessment of children and adolescents because it was designed to be administered within school settings. Its simplicity, short administration time, and its capacity to evaluate multiple individuals simultaneously contribute to its attractiveness. In addition to assessing VO_2_peak, the 20 m shuttle run test provides an evaluation of functional maximal aerobic power (FMAP). FMAP is expressed as the number of completed 1 min stages and is considered another important marker of aerobic fitness [43]. Given its significance as a potential risk factor, there is a strong recommendation to include CRF measurements in both clinical evaluations and population-based studies.

In summary, as demonstrated by the evidence found in the literature, it appears that relying solely on a single anthropometric marker such as BMI provides an incomplete picture of body composition. Integrating markers that capture both central and total obesity is likely to yield a more comprehensive assessment. Furthermore, given the significance of other metrics, such as CRF as potential health markers, clinical evaluations and population-based studies would likely benefit from their inclusion.

Hence, this study aims to achieve three primary objectives: (1) to present the latest standardized normative values for BMI, WC, WHtR, and BSA in Canadian adolescents from Québec; (2) to delineate cardiovascular and/or cardiometabolic risk zones for each of the four anthropometric markers and the two CRF markers; (3) to formulate a comprehensive composite score that concurrently assesses the risk associated with the anthropometric and CRF profile.

## 2. Materials and Methods

### 2.1. Design

This research is an epidemiological cross-sectional study conducted on a large sample size of adolescents aged between 12 and 17 years old. The data utilized in this study were collected by our team through a regional representative school-based survey [43]. The presented data were collected between 2014 and 2017, offering a snapshot of the pre-pandemic COVID-19 situation.

#### 2.1.1. Participants

The present study, conducted in Québec (Canada), involved the participation of 1864 high school students (1008 boys and 856 girls). Participants were selected from a roster of high schools, where 150 schools agreed to take part in the study. To ensure a representative sample, a three-stage sampling approach was employed, targeting a proportional allocation of school boards, schools, and classes [44].

The required sample size for conducting this study was determined through a power analysis using Cohen’s d method, aiming to detect small effects (d < 0.1) at a power (1 − β) of 0.95 for a significance level of (α) of 0.01, using G*Power software (version 3.1.9.4). This analysis determined a necessary sample size of 1564 participants. Using a stratified random sampling method, 12 schools were selected from four cities: Montréal (>2,000,000 inhabitants), Laval (>400,000 inhabitants), Trois-Rivières, and Saguenay (150,000 inhabitants each), representing high and low urban population density. Sociodemographically, Québec stands out as the only Canadian province with a French-speaking majority. While the province boasts an estimated 80% francophone population, the proportion of non-French speakers reaches around 50% in the greater Montréal area. Furthermore, an estimated 25% of Québec’s population originates from immigration, contributing to a more ethnically heterogeneous composition.

To achieve a sample that is representative of urban teenagers in Québec, which represent 85% of the total population, a proportional distribution of students was carried out based on the population of each city. Significant efforts were also invested to ensure the fair and unbiased representation of diverse socioeconomic groups. In Québec, the high school education program spans over a 5-year period and caters to students aged between 12 and 17 years. Both students and parents were informed of the study and given the option to participate or decline. School authorities provided written consent forms. The project received approval from the Institutional Ethical Committee Board of the University of Québec in Chicoutimi (no: 602-225-01).

#### 2.1.2. Anthropometric Markers

All selected tests and measurements were performed using standardized procedures known for their validity and reliability. Anthropometric variables, including body mass (BM), body height (BH), and body mass index (BMI), were collected individually during physical education classes following the procedures recommended by Lohman et al. [45]. WC was collected following the guidelines provided by the National Institutes of Health (NIH), which is also the method proposed by the Canadian Society for Exercise Physiology [46]. Kinesiology interns conducted anthropometric assessments in a separate room adjacent to the gymnasium, ensuring privacy. BM was measured using a Detecto scale (Webb City, MO, USA) accurate to the nearest 0.1 kg, while BH was measured using a SECA model 213 stadiometer (Hamburg, Germany) accurate to the nearest 0.1 cm. WC was assessed using a Gulick anthropometric retractable tape (Wilmington, NC, USA) with a precision of 0.1 cm. If the disparity between two measurements exceeded 1 cm, an additional reading was conducted, and the two measurements with the least discrepancy were averaged to derive the final reading.

The BMI was calculated using the formula $\frac{BM\;(kg)}{BH^{2}(m)}$ and was classified as normal body weight (typical BMI), overweight, or obese according to the classification suggested by Cole et al. [14]. The WHtR was calculated using the formula $\frac{WC\;(cm)}{BH\;(cm)}$. Finally, the BSA was estimated using the method suggested by Mosteller [47], which reads as follows:

$$\mathrm{BSA}\;(m^{2})=\sqrt{\frac{BH\left(cm\right)\times BM(kg)}{3600}}$$

#### 2.1.3. Cardiorespiratory Fitness Markers

CRF was assessed using the 20 m shuttle run test, following the protocol proposed by Léger et al. [40]. This test yields two distinct markers: First, it estimates the FMAP by measuring the number of one-minute stages completed. Second, based on FMAP and participant age, the maximum oxygen consumption (VO_2_peak) is then estimated, which is a more widely used physiological concept.

Testing occurred between 9 am and 3 pm, Monday through Friday, from October to May. Certified kinesiology interns, who had completed a rigorous 45 h training program in anthropometric and cardiorespiratory assessment, meticulously conducted all measurements. Faculty researchers actively involved in the study provided close supervision and support. The 20 m shuttle run test took place indoors in high school gymnasiums. To maximize effort, participants received verbal encouragement throughout the test. To ensure individual attention, no more than 20 participants were assessed simultaneously.

#### 2.1.4. Data Exclusions

Before curve smoothing, outliers and participants whose weight-for-height values were below the 0.135th percentile or above the 97.7th percentile, which is considered “unhealthy” by the WHO guidelines [48], were excluded. Furthermore, only students with measured WC were included. This resulted in excluding 55 participants (3.0%), leaving a final sample size of 1809 students.

#### 2.1.5. Statistical Analysis

Descriptive statistics are presented as mean ± standard deviation (SD) with 95% confidence intervals (CI). Cohen’s d effect sizes were calculated for intergroup comparisons. The normality of each variable was assessed using the Shapiro–Wilk test. For non-normal distributions, a Box-Cox transformation was applied. Curves were generated using the Box-Cox power exponential approach employing cubic splines, as recommended by the WHO in 2006 [48].

Outliers were detected by applying the method proposed by Hoaglin and Iglewicz [49] while percentile values were computed using the LMS method as suggested by Cole and Green [50]. A detailed description of the methodology employed in this study has been previously documented [43,51].

For nearly two decades, numerous studies have advocated for using the 85th percentile of BMI as a benchmark to identify children and adolescents aged 5 to 19 who are overweight or obese [1,17,29,32,36]. In the case of markers such as WC, WHtR, and BSA, it is unclear if the 85th percentile should be applied. To investigate this question, we examined adolescents in our sample whose BMI corresponded to ≥85th percentile and analyzed the corresponding Z-scores for the three other anthropometric markers. We found that the associated value for BMI (85th percentile) is actually aligned more closely to the 80th percentile, indicating a more stringent threshold. Similar findings have been reported elsewhere [10,52], supporting the need for distinct thresholds for each marker, which are delineated as follows:

BMI:

Low-risk zone < 85th percentile
High-risk zone = 85th–95th percentile
Very-high-risk zone > 95th percentile

WC, WHtR, and BSA:

Low-risk zone < 80th percentile
High-risk zone = 80th–95th percentile
Very-high-risk zone > 95th percentile

Furthermore, a warning zone created for values between the 60th and 80th percentile to provide an initial alert, even though this range does not present an immediate risk. Indeed, previous research [14] indicates that the buffer zone for BMI is defined in an arbitrary manner, encompassing percentiles ranging from the 60th to the 85th percentile, instead of being limited to the 80th percentile as previously stated.

For the CRF, the risk zones were determined based on the quartiles presented below, which have also been used in prior research [38,53]:

Low-risk zone > 50th percentile
High-risk zone = 50th–25th percentile
Very-high-risk zone < 25th percentile

A composite score was developed to assess cardiovascular health risk by combining four anthropometric markers (BMI, WC, WHtR, and BSA) with one of two cardiovascular risk factor markers: VO_2_peak or FMAP. The selected markers represent three distinct risk factors: overall obesity (BMI and BSA), central obesity (WC and WHtR), and low CRF (VO_2_peak and FMAP). Each factor receives equal weight in calculating the composite score. To standardize the values of each marker, raw scores were first transformed into Z-scores and then expressed as percentiles, as previously outlined. This assigned each participant a risk zone (low-risk, high-risk, or very-high-risk) for each marker. It should be noted that the warning zone is part of the low-zone risk, as explained previously.

The regression equations were derived using forward multiple linear regression techniques. Additionally, various statistical measures were calculated to assess model performance, including the standard error of the estimate (SEE), standard error of the mean (SEM), mean absolute error (MAE), root mean squared error (RMSE), variance inflation factor (VIF), and coefficient of determination (R^2^). The regression equations presented establish cutoff points for interpreting cardiometabolic risk. Scores exceeding the 70th percentile indicate high risk, while scores surpassing the 85th percentile signify very-high risk. These thresholds were derived from the cumulative percentile averages of WC, BMI, VO_2_peak, and FMAP. These composite thresholds differ from individual marker thresholds because they account for the combined influence of all considered factors.

Due to variations in WC assessment methods found in the literature (i.e., WHO vs. NIH), Patry-Parisien et al. [54] proposed the following equations to standardize the measurements and enhance their comparability:

Boys: WC corrected = −0.8991 + (WC_WHO × 1.01829) + (age × 0.05164)

Girls: WC corrected = −0.70299 + (WC_WHO × 1.01891) + (age × 0.12297)

## 3. Results

Table 1 presents descriptive statistics for BM, BH, and CRF characteristics across age and sex. While VO_2_peak values naturally decline with age for both sexes, comparing consecutive years (e.g., 12 vs. 13) does not reveal statistically or clinically significant differences. However, analyzing the entire adolescence period reveals a statistically significant and clinically meaningful decline in VO_2_peak (*p* = 0.0001). In boys, VO_2_peak drops from 44.8 mL·kg^−1^·min^−1^ at age 12 to 40.9 mL·kg^−1^·min^−1^ at age 17, representing a moderate effect size (Cohen’s d = 0.63). For girls, the decline is even steeper, falling from 41.6 mL·kg^−1^·min^−1^ to 33.9 mL·kg^−1^·min^−1^, with a large effect size (Cohen’s d = 1.63). Interestingly, the number of 1 min stages completed follows a different pattern, peaking at age 16 for boys and from age 15 for girls.

Table 2 displays anthropometric characteristics of WC, BMI, WHtR, and BSA categorized by sex and age. Notably, values for all four markers increase with age for both sexes, except for WHtR, which tends to remain relatively stable throughout adolescence.

**Figure 1:**
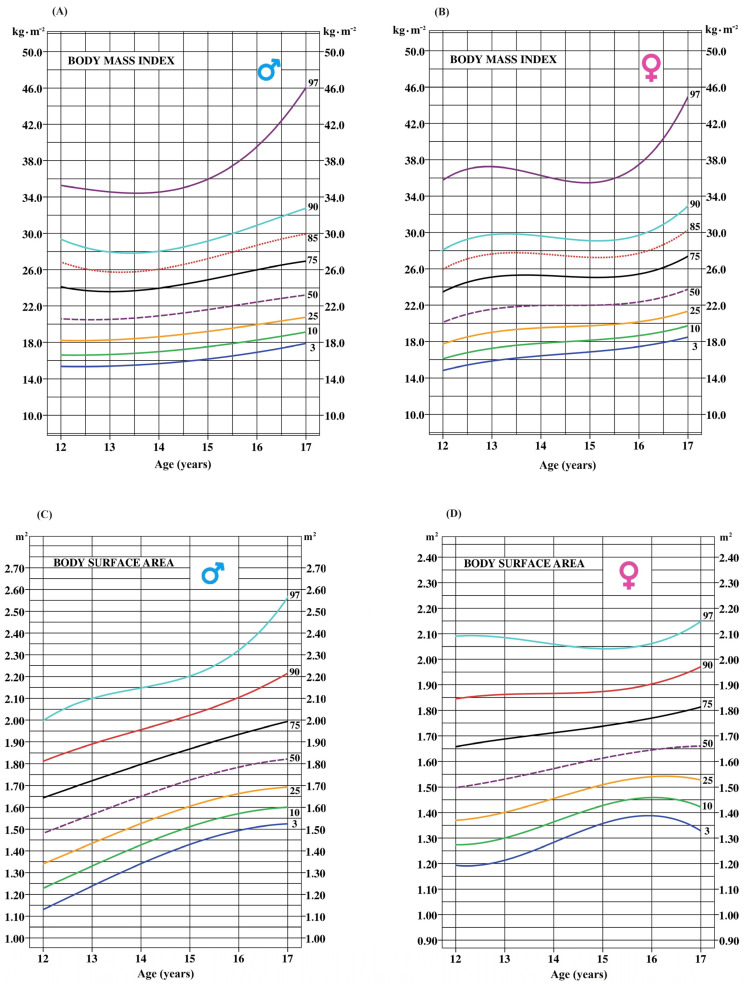
Age-specific smoothed percentile curves for BMI and BSA for boys (**A**,**C**) and girls, respectively (**B**,**D**).

**Figure 2:**
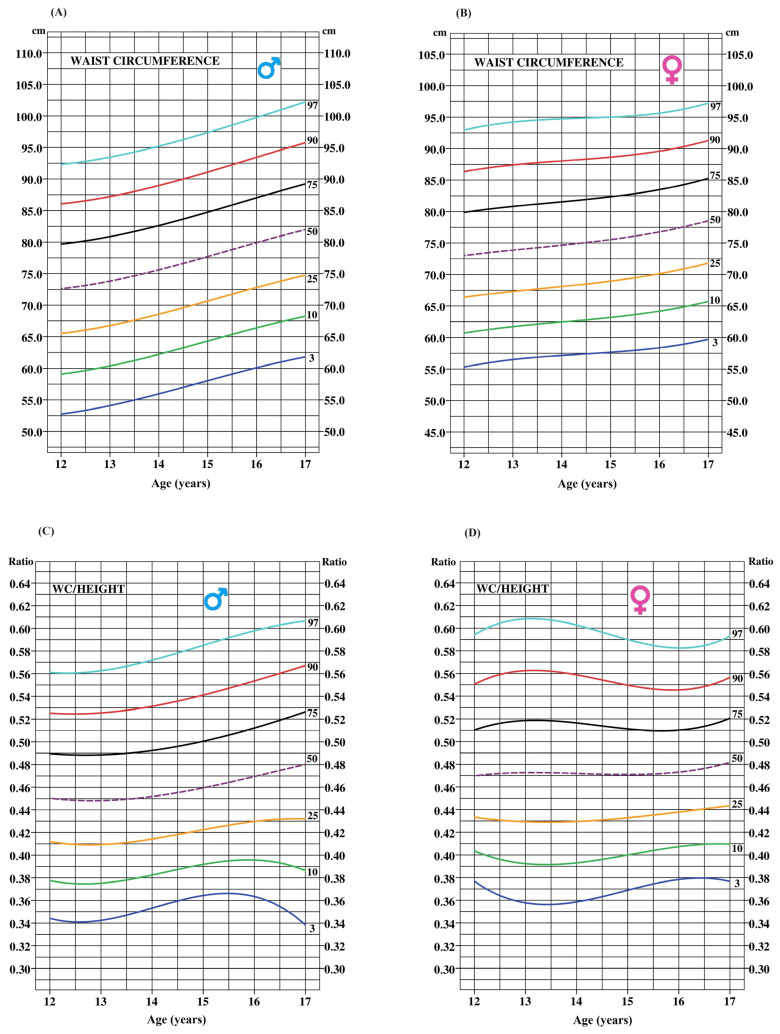
Age-specific smoothed percentile curves for WC and WHtR for boys (**A**,**C**) and girls, respectively (**B**,**D**).

Figure 1 and Figure 2 display smoothed age-specific percentile curves for BMI, BSA, WC, and WHtR (A and C for boys; B and D for girls). The dotted line represents the median curve for each marker.

Table 3 and Table 4 offer a valuable tool for identifying specific percentile values of each anthropometric marker categorized by age and sex. These tables present the data in 10-percentile intervals from 10th to 90th, along with the 5th and 95th percentiles, which hold clinical significance. It is worth noting that the LMS method parameters have also been included, so that other percentile values can be calculated if required.

**Figure 3:**
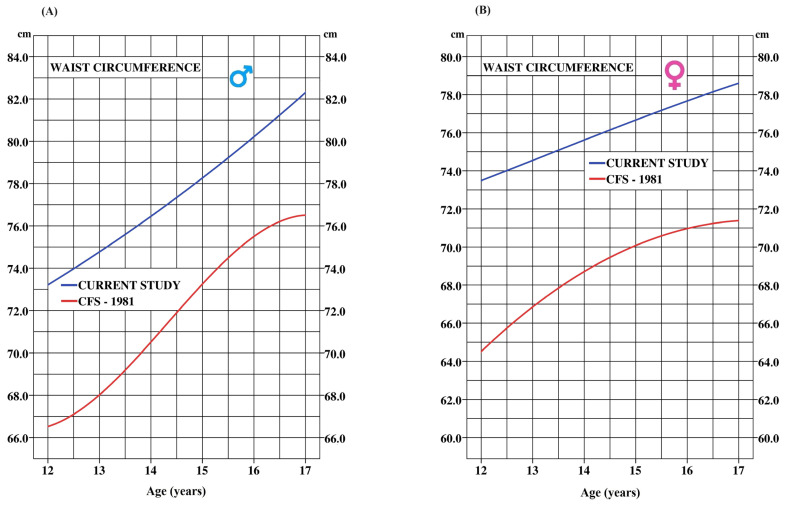
Secular trend for waist circumference (WC) between the current study (Blue) and adjusted data from the 1981 Canadian Fitness Survey (CFS; Red)). Boys (**A**) and girls (**B**).

Figure 3 depicts the secular trend in WC from 1981 onwards, carefully adjusted for methodological differences between the two studies. In the 2000s, the measurement of WC was assessed using the method proposed by the National Institutes of Health (NIH), whereas the method used previously was that suggested by the World Health Organization (WHO). To ensure data comparability, a correction factor recommended by Patry-Parisien et al. [54] was applied.

Despite data normalization aiming to minimize differences between the two studies, statistically significant disparities persist between the two-time frames for each age group (*p* = 0.0001) in both boys and girls (Table 5). Compared to their 1981 counterparts, boys in 2017 exhibited a notable increase in WC (5.8 cm), and the same trend held true for girls (7.4 cm increase). Interestingly, the Canadian Fitness Survey (CFS) curves from 1981 suggest a plateau in WC around age 16 for both sexes (55). In contrast, the current study reveals a continued rise in WC beyond this age.

### Risk Stratification

**Figure 4:**
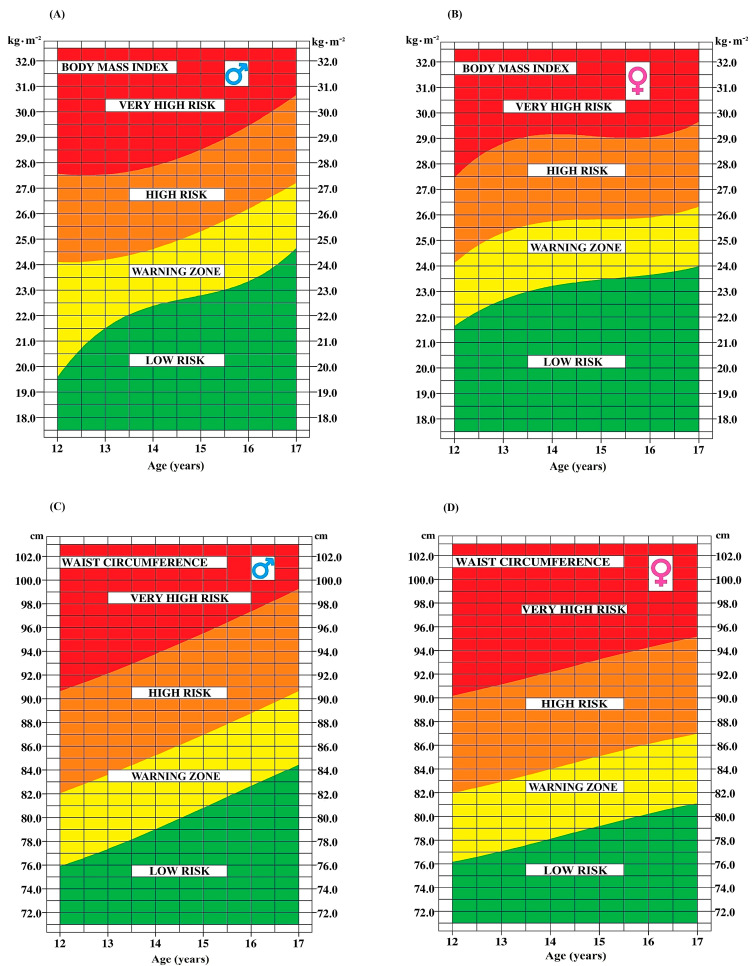
Age-dependent determination of health risk zones for body mass index and waist circumference in boys (**A**,**C**) and girls (**B**,**D**).

**Figure 5:**
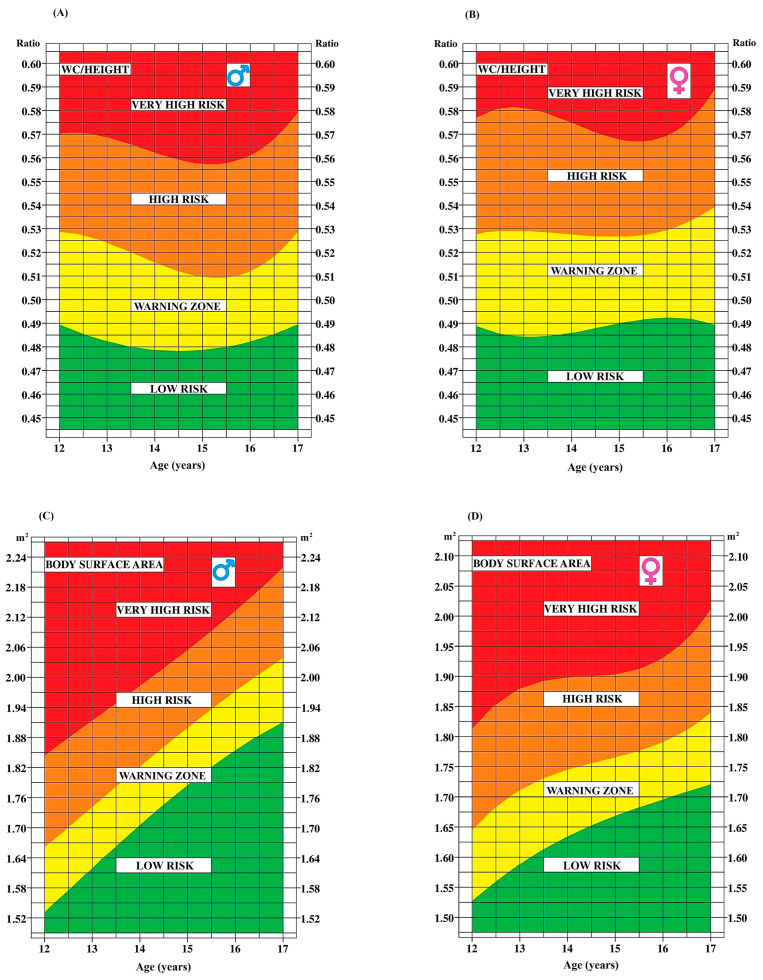
Age-dependent determination of health risk zones for waist-to-height ratio and body surface area in boys (**A**,**C**) and girls (**B**,**D**).

**Figure 6:**
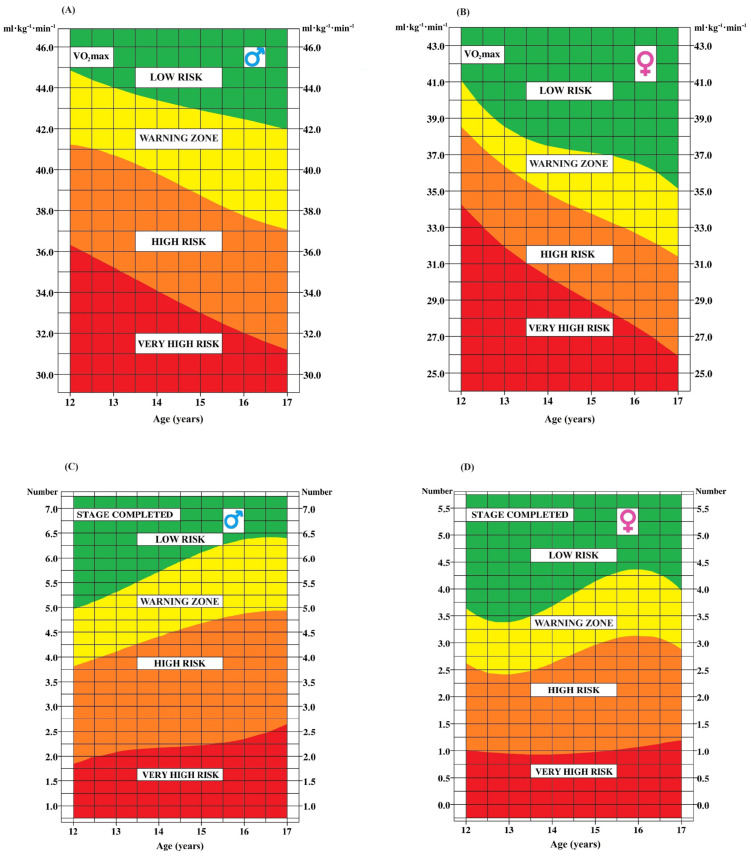
Age-dependent determination of health risk zones for VO_2_peak and the number of stages completed in the 20 m shuttle run test in boys (**A**,**C**) and girls (**B**,**D**).

Beyond presenting anthropometric measurements, this paper also provides insightful tools to assess health risks associated with these values. Figure 4, Figure 5 and Figure 6 provide a convenient and efficient way to visualize the health risk zones for each anthropometric and CRF marker, categorized by age and sex (A for boys and B for girls). The inclusion of the yellow area, denoting a warning zone, suggests a potential trajectory towards higher risk zones. It is useful to interpret it as a warning sign, even in the absence of immediate threat.

Table 6 provides a clear and concise summary of health risks associated with each of the six markers, categorized into three distinct zones: low-risk, high-risk, and very-high-risk. This allows clinicians to quickly identify their patients’ risk level based on raw marker values. Importantly, BMI risk zones were established using age- and sex-specific recommendations from the WHO. For the remaining markers, the risk zones were determined using Z-scores derived from our sample population, as there is currently no international consensus on the specific values for risk zones.

Table 7 compares the measured and predicted percentile values of the composite scores. Due to collinearity issues among some variables, we selected an optimal model that utilizes only four markers: WC, BMI, VO₂peak, and FMAP. This model is pragmatic as it integrates some of the most cited markers found in the relevant literature. To enhance the model’s versatility and accommodate situations where CRF assessment might not involve the 20 m shuttle run test, a second comparison was performed excluding FMAP (number of 1 min stage). Both equations boast coefficients of determination exceeding 90%, indicating that each model effectively explains a high proportion of the shared variance. Notably, the four-marker equation exhibits no statistically significant discrepancies between measured and predicted values. While the equation omitting FMAP shows a significant difference (*p* = 0.016), Cohen’s d coefficient reveals this difference to be clinically insignificant (less than 0.5 percentile point). Consequently, we can confidently conclude that the two models perform equivalently.

Along the same lines, Table 8 presents two models, one with and one without the FMAP marker. Although the model with four markers appears slightly more precise, the two equations are completely compatible with the objective of early detection of individuals at risk. Firstly, the high coefficients of determination (r^2^~0.89) indicate that the independent variables account for nearly 90% of the explained variance. This result is particularly interesting, suggesting a strong explanatory power of the model. Secondly, the VIF values indicate that the two equations are unlikely to contain collinearity bias since each of the markers included in these two models display values well below the critical threshold of 10. Finally, based on RMSE values and more particularly the MAE, it can be concluded that the two models are valid and display satisfactory precision in determining the cardiometabolic risk of adolescents.

Table 9 reveals that, based on individual analysis of the four anthropometric markers, nearly 25% of the adolescents in this study exhibit a high or very-high risk of developing cardiometabolic problems in the short or long term. Considering CRF markers alongside anthropometric markers (composite score) increases this percentage to nearly 33%. This increase is primarily attributed to the significant contribution of CRF markers, which exceed 50% in both boys and girls who fall within the high- or very-high-risk zones for VO_2_peak and for FMAP. This percentage reaches its peak at 17 years old, with boys facing a risk of around 55% and girls exceeding 60%.

## 4. Discussion

This study provided a valuable opportunity to revisit and document various understudied anthropometric markers in the Canadian adolescent population. One such marker is WC, which has only recently received attention, with the first publication of normative values in Canada occurring in 2004. Notably, the data used to establish these norms originated from a survey conducted much earlier, in 1981 [55]. Consequently, considering the observed changes in obesity rates over the past few decades, the 2004 normative values likely underestimate current WC in Canadian adolescents. This is further supported by a 2010 study, which compared data from 1981 with 2007–2009, documenting increases in WC of 4.2 cm and 6.7 cm for boys and girls, respectively [18]. Conversely, the present study reveals an even greater increase, with values reaching 5.8 cm in boys and 7.4 cm in girls, suggesting a sustained secular trend. Remarkably, the observed trend in WC appears to exhibit a distinct trajectory compared to that of BMI.

Indeed, a recent investigation conducted by our research team within the same demographic population reported a plateau in overweight and obesity rates between 2004 and 2017 based on BMI assessment [51]. These contrasting trends in the phenotypic evolution of central and general obesity markers highlight the significance of considering both dimensions in assessing obesity. Furthermore, this study used a composite score to identify cardiometabolic health risk in young individuals. This approach helps alleviate the constraints associated with relying on a single marker. Furthermore, the establishment of a “warning zone” enables clinicians to prioritize specific markers that, while not indicative of immediate danger, warrant more frequent and vigilant monitoring. Finally, the inclusion of CRF markers into our model enhances the likelihood of early detection of adolescents who may be at risk of developing cardiometabolic issues.

### 4.1. WHtR and BSA as Cardiometabolic Risk Markers

While BMI and WC are widely used to assess current and future possibility of developing cardiometabolic conditions, it is important to acknowledge the relevance of other anthropometric markers as well. Specifically, WHtR and BSA are markers that also have been associated with cardiometabolic risk [24,25,26,33,34]. Including these markers can enhance the reliability of the information provided by BMI and WC alone. Notably, retrieving this supplementary information is straightforward since WHtR and BSA are derived from the same measured variables, namely BM, BH, and WC. To our knowledge, only one Canadian study has provided normative values for WHtR, and this research was conducted nearly a decade ago [52]. In addition, the present study is the first to provide normative values for BSA in a large sample of Canadian adolescents. Therefore, publishing new (BSA) or updated normative values (WHtR) will likely benefit public health leaders and clinicians.

### 4.2. Inclusion of CRF Markers

The inclusion of physiological markers represents a significant advancement in cardiometabolic risk assessment. The addition of markers such as VO_2_peak or the number of 1 min stages completed (FMAP) in the 20 m shuttle run test provides independent insights into adolescents’ health, leading to a more comprehensive characterization of their risk profile. Moreover, integrating this physiological dimension aligns directly with the American Heart Association’s recommendation, as outlined in their scientific statement, which promotes cardiorespiratory fitness (CRF) as a “vital sign” that should be routinely monitored in clinical practice [36]. In this model, low CRF assumes a central role in the estimation of cardiometabolic risk. In our study, CRF could either be determined using VO_2_peak or the number of stages completed. The choice between the two markers is left to the user, and this was chosen for pragmatic reasons. While diverse methods exist to measure VO_2_peak, many are resource-intensive. Treadmills, bicycle ergometers, and expired gas analyzers can be costly, time-consuming, and require specialized expertise. Fortunately, simpler and more cost-effective field tests still yield valid results. Therefore, our model accepts VO_2_peak values obtained through diverse methodologies (not necessarily the 20 m shuttle run test), promoting both versatility and accessibility.

Unlike anthropometric markers readily calculated from BM, BH, and WC and easily included in the proposed model, the number of 1 min stages completed, serving as an indicator of an individual’s FMAP, can only be obtained through the 20 m shuttle run test. Consequently, the obligatory inclusion of both CRF markers would restrict the possibility of obtaining a composite score, leading to the option of including only one of these two markers. In fact, the current study demonstrates the feasibility of developing an accurate and reliable model using a limited number of markers to predict the cardiometabolic risk among adolescents. While we advocate for the use of both CRF markers (Equation (1)), employing solely the VO_2_peak value offers a viable alternative for overall risk assessment with a marginal impact of only ~−6%.

### 4.3. Individual and Composite Scores as Cardiometabolic Risk Markers

The current study provides a more complete assessment of cardiometabolic risk as opposed to studies using individual markers. However, establishing an overall risk classification that encompasses all markers simultaneously presents a challenge. The composite score offers the benefit of summarizing the risk when analyzing all markers concurrently. It is important to acknowledge that both the individual risk zones and the final composite score have inherent limitations due to their arbitrary nature. However, the delineation of the various risk zones largely mirrors the rationale used by the WHO to determine the BMI risk zones. Consequently, each risk zone was established using corresponding BMI values. For instance, to determine WC risk, we first identified the raw values corresponding to the 85th percentile for BMI. Subsequently, the raw score was compared with the corresponding Z-score. Thus, for anthropometric variables, it has been determined that the risk zone corresponding to the 85th percentile for BMI aligns with the 80th percentile for WC, WHtR, and BSA. Moreover, previous studies have already recognized the value aligned with the 80th percentile as the most appropriate for adolescents concerning WC [10], which is consistent with our findings. This approach, adopted in previous studies [4,29], acknowledges the unique characteristics of the target population instead of applying a universal cutoff point. In this context, we have forsaken the “conventional” cutoffs for WC, WHtR, and BSA in favor of thresholds more tailored to the Canadian population. Nonetheless, our model can be adapted for other populations based on the same rationale. This more stringent 80th percentile cutoff enhances the sensitivity of the screening tool by minimizing the likelihood of overlooking adolescents who may be at risk of health issues.

Our regression analysis revealed collinearity issues among the anthropometric markers, particularly between WC and WHtR. We prioritized retaining WC due to its wider use and more extensive research base. Following the same rationale, BSA was excluded in favor of BMI. However, excluding WHtR and BSA from the model does not diminish their clinical value. From an individual analysis, the BSA and WHtR indices can bolster the clinician’s evaluation by confirming the trends observed through BMI or WC measurements. Consequently, these two parameters can supplement the clinician’s assessment in addition to BMI or WC values. For instance, when both BMI and BSA categorize an adolescent into the same risk group, it indicates that the risk linked with general obesity is supported by two indicators rather than solely one. In essence, the incorporation of BSA and WHtR significantly enhances the clinical evaluation, facilitating a more precise diagnosis and personalized patient management, while providing a more complete vision of their cardiometabolic status and the associated risk of obesity-related complications.

CRF markers represent a distinct facet of cardiometabolic risk that needs to be considered. While a consensus on risk values for adolescent health remains elusive, some researchers have proposed cutoff value below 42 mL·kg^−1^·min^−1^ for boys and 35 mL·kg^−1^·min^−1^ for girls, albeit with certain variations [36,38,43]. While these values account for sex differences, no studies have specifically addressed age-related cutoff values. Interestingly, during the 1980s, a single cutoff point was established for children and adolescents aged 6 to 17, particularly among boys, due to the remarkable consistency of relative VO_2_peak, around 50 mL·kg^−1^·min^−1^, throughout this period of physical growth [40]. However, current evidence clearly demonstrates that relative VO_2_peak values tend to decline with age in both boys and girls [41,42,43]. This growing trend, spanning several decades, is indicative of the shift observed in today’s adolescents who are forsaking physical activities in favor of adopting more sedentary behaviors, such as engaging in activities like playing video games, for instance. Thus, this age-related decline in VO_2_peak must be considered when estimating health risk. For instance, if the cutoff point is set at 42 mL·kg^−1^·min^−1^ for 12-year-old boys, considering their subsequent decline in VO_2_peak, these same boys are likely to fall below this threshold at the age of 13. Thus, in this particular case, the cutoff point of 42 mL·kg^−1^·min^−1^ may provide a false sense of security for an individual of this age.

Given the observed decline, a higher cutoff threshold would be more appropriate for 12-year-olds compared to 13-year-olds, considering their inherently higher median VO_2_peak. Based on this model, we note that more than 50% of adolescents in our sample are at high CRF risk. It is worth highlighting that this risk reaches its peak at the age of 17, with a value of 57.3% for boys and 65.8% for girls, corroborating recent published data [43]. These findings are undoubtedly concerning and warrant public health attention. It is crucial to take immediate measures to motivate adolescents to embrace a more active lifestyle, particularly older individuals who are already at a heightened risk of imminent cardiometabolic problems. One potential solution involves reevaluating the allocation of physical education (PE) within school curriculums. The significant reduction in PE minutes observed in Québec over the past three decades coincides with the decline in VO_2_peak and FMAP values, suggesting a potential link that merits further investigation [43].

Regarding the number of 1 min stages completed (FMAP), as far as we know, only one prior study has investigated its association with cardiometabolic risk [38]. In general, the reported values align with the results of the present study, showing similar magnitudes when considering values per year of chronological age. Even though the data originates from different populations, which could account for certain variations, it is worth noting that both studies were conducted during the same timeframe (2016 vs. 2017 for the present study), thereby minimizing the potential impact of the secular trend.

Therefore, the inclusion of a marker directly focusing on adolescents’ functional capacity, like FMAP, undoubtedly constitutes a significant contribution to their cardiometabolic risk assessment. While the data primarily originates from the province of Québec, which accounts for slightly less than 25% of the Canadian population, the proposed model can serve as a valuable reference for other regions within Canada and potentially worldwide. In the absence of regional data, the values from this study can serve as a provisional risk assessment tool until region-specific values becomes available. FMAP represents a cardiometabolic risk marker that is equally, if not more, significant compared to VO_2_peak. It holds the advantage of being less influenced by anthropometric characteristics in comparison to relative VO_2_peak. Consequently, FMAP’s interpretation is simpler, both for adolescents and for healthcare professionals. Particularly during growth spurts, when considering available data, FMAP should take precedence over VO_2_peak as the primary marker for assessing cardiometabolic risk. Nonetheless, for cardiometabolic composite risk assessment, regression Equation (2) demonstrates the feasibility of using VO₂peak alone when FMAP data is unavailable. Despite the absence of FMAP data, VO_2_peak alone can be a sufficient metric for assessing composite cardiometabolic risk (regression Equation (2)) without compromising on validation and accuracy, which remain excellent.

### 4.4. The Relative Contribution of Marker Categories to Cardiometabolic Risk: A Nuanced Analysis

Based on both the existing literature and our study’s findings, it remains challenging to ascertain whether anthropometric markers (BMI, WC, BSA, and WHtR) or characteristics related to cardiorespiratory fitness markers (VO_2_peak and FMAP) exert a more significant influence on the development of cardiometabolic issues. In the absence of a clear consensus, we assigned equal weight to both of these marker categories. Nonetheless, our findings highlight a significant disparity in the risk distribution across marker categories among Canadian adolescents. Specifically, approximately 25% of adolescents displayed an elevated risk solely based on anthropometric markers, contrasting with a roughly 50% risk for CRF markers. The composite score emerges as an important tool in contextualizing these results by adjusting the overall risk assessment to account for the interaction between the two marker groups. A low risk in one group can mitigate the overall risk, particularly when another group presents a high risk. In fact, when considered in isolation, anthropometric markers likely tend to minimize cardiometabolic risk, while CRF markers may tend to overestimate it. Therefore, the composite score offers a more refined and holistic approach to evaluating cardiometabolic risk in adolescents by incorporating the synergistic effects between anthropometric and CRF markers. Thus, rather than having to interpret each marker individually to decipher an overall cardiometabolic profile, the composite score streamlines the process by enabling interpretation based on only three values: low risk ≤ 69th percentile; high risk 70–84th percentile; very-high risk ≥ 85th percentile. This comprehensive approach ultimately leads to a more nuanced interpretation of individual risk profiles (see Supplementary Materials).

### 4.5. Limitations and Strengths

This study has several limitations. First, the cross-sectional design restricts the ability to draw causal inferences. Second, the estimation of VO_2_peak values instead of direct measurement introduces inherent uncertainties. Third, the analysis employs anthropometric markers derived from field measurements. These measurements may not consistently achieve the optimal level of precision required for drawing robust inferences, compared to those obtained from direct measures. Fourth, while the sample represents Canadian adolescents living in Québec, generalizing the findings to other regions requires caution. Furthermore, the associations between cardiometabolic risks and the measured markers are primarily based on existing literature, limiting the study’s ability to establish causality. However, this study also boasts notable strengths. The substantial participant size (N = 1864) provides a valid representation of Québec adolescents. Additionally, the stratification of several criteria, including age groups, sex, ethnicity, and socio-economic status, enhances the representativeness of the evaluated population. Beyond its user-friendly characteristics, the proposed model provides a level of validity and precision that ensures reliable data interpretation. Finally, despite the weaknesses mentioned above, all employed markers in this study are well established as valid and reliable measures, displaying acknowledged associations with cardiometabolic risks.

## 5. Conclusions

This study stands out as one of the few that has highlighted the association between a combination of anthropometric and CRF markers and their relationship with the probability of developing cardiometabolic conditions. Notably, data on WC and WHtR is particularly limited in the Canadian context. Furthermore, to our knowledge, this study is the first in Canada to incorporate BSA and present its standardized normative values, offering an additional tool for identifying adolescents at risk of cardiometabolic pathologies. Furthermore, the establishment of risk categories across multiple domains is also a novel contribution. Not only do these zones enable the measurement of risk on a marker-by-marker basis, our model also facilitates an overall evaluation of cardiometabolic risk by simultaneously considering anthropometric and CRF markers through a composite score. Over one-third of Canadian adolescents in Québec, as evidenced by this study, exhibit risk profiles indicating a high to very-high likelihood of developing cardiometabolic problems in the short or long term. This highlights a significant public health concern that requires immediate attention. Therefore, we believe that this model holds significant potential for public health surveillance purposes as well as in clinical settings for individual assessment.

## Figures and Tables

**Table 1:** Descriptive statistics for body mass, body height, and cardiorespiratory fitness variables in adolescents aged 12 to 17 years.

Age	N	Body Mass (kg)	CI 95%	Body Height (cm)	CI 95%	VO_2_max mL·kg^−1^·min^−1^	CI 95%	Stages (Number)	CI 95%
		Mean ± SD		Mean ± SD		Mean ± SD		Mean ± SD	
Boys									
12	128	48.2 ± 9.9	46.4–49.9	155.1 ± 8.6	153.6–156.6	44.8 ± 5.3	43.8–45.7	5.1 ± 2.0	4.8–5.5
13	223	54.8 ± 13.1	53.1–56.5	161.3 ± 9.0	160.1–162.5	43.8 ± 4.9	43.2–44.5	5.3 ± 1.9	5.1–5.5
14	173	57.6 ± 10.2	56.1–59.2	166.5 ± 7.7	165.3–167.7	44.6 ± 6.8	43.6–45.6	6.2 ± 2.5	5.8–6.5
15	205	62.9 ± 11.1	61.4–64.5	170.6 ± 6.6	169.6–171.5	43.1 ± 6.9	42.1–44.1	6.2 ± 2.5	5.9–6.5
16	161	66.4 ± 11.7	64.5–68.3	172.7 ± 7.6	171.6–173.9	42.7 ± 7.2	41.6–43.8	6.6 ± 2.5	6.2–7.0
17	93	68.9 ± 11.6	66.4–71.4	173.5 ± 7.8	171.9–175.1	40.9 ± 7.2	39.4–42.4	6.6 ± 2.4	6.1–7.0
Girls									
12	161	47.9 ± 9.4	46.4–49.4	154.9 ± 6.3	153.9–155.9	41.6 ± 4.4	40.9–42.3	3.7 ± 1.7	3.5–4.0
13	211	51.6 ± 10.6	50.2–53.1	157.5 ± 6.1	156.7–158.3	39.6 ± 4.7	38.9–40.2	3.7 ± 1.7	3.5–3.9
14	143	55.3 ± 10.1	53.6–57.0	158.3 ± 6.2	157.2–159.3	37.4 ± 4.2	36.7–38.1	3.5 ± 1.6	3.2–3.7
15	117	56.9 ± 8.7	55.3–58.6	161.5 ± 6.7	160.3–162.7	38.3 ± 5.6	37.3–39.4	4.6 ± 1.9	4.2–4.9
16	128	58.8 ± 9.2	57.2–60.4	163.0 ± 7.0	161.8–164.3	36.2 ± 5.9	35.1–37.2	4.3 ± 2.1	4.0–4.7
17	71	59.7 ± 9.9	57.3–62.1	162.7 ± 7.8	160.8–164.5	33.9 ± 5.4	32.7–35.2	4.1 ± 1.8	3.7–4.5

Age = years; SD = standard deviation; CI 95% = confidence interval at 95% level; Stages = number of one-minute stage completed in the 20 m shuttle run test.

**Table 2:** Anthropometric characteristics for waist circumference (WC), body mass index (BMI), waist-to-height ratio (WHtR), and body surface area (BSA) in Québec (Canada) adolescents.

Age (Years)	N	WC (cm)	CI 95%	BMI (kg·m^−2^)	CI 95%
Boys					
12.0–12.9	125	73.2 ± 10.6	71.4–75.1	20.7 ± 4.2	19.9–21.4
13.0–13.9	223	74.8 ± 10.3	73.5–76.2	20.9 ± 4.0	20.4–21.5
14.0–14.9	170	76.4 ± 10.6	74.8–78.0	21.2 ± 3.9	20.6–21.8
15.0–15.9	206	78.3 ± 10.3	76.9–79.7	21.9 ± 4.1	21.4–22.5
16.0–16.9	157	80.2 ± 10.2	78.5–81.8	22.5 ± 4.4	21.8–23.2
17.0–17.9	92	82.3 ± 10.1	80.2–84.3	23.5 ± 4.4	22.6–24.5
Girls					
12.0–12.9	160	73.5 ± 10.1	72.0–75.1	20.8 ± 4.3	20.1–21.4
13.0–13.9	211	74.5 ± 10.1	73.2–75.9	21.1 ± 4.1	20.5–21.7
14.0–14.9	144	75.7 ± 9.8	74.1–77.3	23.0 ± 4.8	22.2–23.8
15.0–15.9	116	76.6 ± 10.1	74.8–78.5	22.1 ± 3.4	21.4–22.7
16.0–16.9	130	77.7 ± 10.0	76.0–79.4	22.8 ± 4.0	22.0–23.4
17.0–17.9	72	78.6 ± 10.0	76.2–81.0	23.0 ± 4.1	22.0–23.9
Boys					
Age (Years)	N	WHtR (Ratio)	CI 95%	BSA (m^2^)	CI 95%
12.0–12.9	128	0.47 ± 0.06	0.46–0.49	1.47 ± 0.22	1.44–1.51
13.0–13.9	223	0.46 ± 0.06	0.45–0.47	1.57 ± 0.22	1.54–1.60
14.0–14.9	172	0.46 ± 0.06	0.45–0.47	1.65 ± 0.19	1.62–1.68
15.0–15.9	208	0.46 ± 0.06	0.45–0.47	1.74 ± 0.20	1.71–1.77
16.0–16.9	156	0.46 ± 0.06	0.45–0.47	1.79 ± 0.20	1.76–1.83
17.0–17.9	93	0.48 ± 0.07	0.46–0.49	1.85 ± 0.22	1.81–1.90
Girls					
12.0–12.9	162	0.48 ± 0.06	0.47–0.49	1.48 ± 0.21	1.44–1.51
13.0–13.9	212	0.47 ± 0.06	0.46–0.48	1.52 ± 0.20	1.50–1.55
14.0–14.9	144	0.48 ± 0.07	0.47–0.50	1.60 ± 0.21	1.57–1.64
15.0–15.9	117	0.47 ± 0.06	0.46–0.48	1.61 ± 0.15	1.59–1.64
16.0–16.9	131	0.48 ± 0.05	0.47–0.49	1.66 ± 0.18	1.62–1.69
17.0–17.9	72	0.49 ± 0.06	0.47–0.50	1.67 ± 0.21	1.62–1.72

*N* = number of participants; Variables = mean ± standard deviation; CI 95% = confidence interval at 95%.

**Table 3:** Smoothed percentile standards for body mass index (BMI) and waist circumference (WC) according to age (years) and sex in Québec adolescents.

BMI (kg·m^−2^)					Percentiles										
Age	N	L	M	S	P_5_	P_10_	P_20_	P_30_	P_40_	P_50_	P_60_	P_70_	P_80_	P_90_	P_95_
Boys (*N* = 983)															
12.0–12.9 years	128	−1.81	20.6	0.203	15.9	16.6	17.7	18.7	19.6	20.6	21.7	23.2	25.3	29.3	34.4
13.0–13.9 years	223	−1.12	20.5	0.191	15.7	16.5	17.7	18.6	19.6	20.5	21.5	22.8	24.5	27.3	33.0
14.0–14.9 years	171	−1.71	21.0	0.184	16.5	17.2	18.3	19.2	20.1	21.0	22.0	23.3	25.1	28.4	32.2
15.0–15.9 years	208	−1.02	21.5	0.187	16.5	17.3	18.6	19.6	20.5	21.5	22.6	23.8	25.5	28.3	31.1
16.0–16.9 years	161	−1.85	22.5	0.196	17.5	18.3	19.5	20.5	21.5	22.5	23.7	25.2	27.4	31.5	36.7
17.0–17.9 years	92	−2.19	23.2	0.187	18.3	19.1	20.3	21.2	22.2	23.2	24.4	25.9	28.1	32.6	38.7
Girls (*N* = 838)															
12.0–12.9 years	162	−1.51	20.3	0.207	15.4	16.2	17.4	18.4	19.3	20.3	21.4	22.9	24.9	28.5	32.7
13.0–13.9 years	213	−1.26	20.9	0.194	16.0	16.8	18.0	19.0	19.9	20.9	22.0	23.3	25.1	28.2	31.4
14.0–14.9 years	144	−1.54	22.6	0.209	17.2	18.1	19.3	20.4	21.5	22.6	23.9	25.5	27.7	31.9	36.9
15.0–15.9 years	117	−1.32	22.0	0.154	17.7	18.5	19.5	20.4	21.2	22.0	22.9	24.0	25.4	27.7	29.9
16.0–16.9 years	131	−2.00	22.0	0.180	17.4	18.2	19.3	20.2	21.1	22.0	23.1	24.4	26.4	30.0	34.5
17.0–17.9 years	71	−2.27	23.9	0.178	19.0	19.9	21.0	22.0	22.9	23.9	25.1	26.5	28.7	33.0	38.7
WC (cm)					Percentiles										
Age	N	L	M	S	P_5_	P_10_	P_20_	P_30_	P_40_	P_50_	P_60_	P_70_	P_80_	P_90_	P_95_
Boys (*N* = 976)															
12.0–12.9 years	125	1.02	72.8	0.145	55.4	59.2	63.9	67.3	70.1	72.8	75.5	78.3	81.7	86.3	90.1
13.0–13.9 years	224	1.04	73.3	0.141	56.2	60.0	64.6	67.9	70.7	73.3	75.9	78.7	82.0	86.5	90.2
14.0–14.9 years	171	0.98	75.8	0.139	58.5	62.3	66.9	70.3	73.1	75.8	78.5	81.3	84.7	89.3	93.2
15.0–15.9 years	206	0.99	78.3	0.134	61.1	64.9	69.5	72.8	75.6	78.3	81.0	83.8	87.1	91.8	95.6
16.0–16.9 years	157	1.01	79.2	0.132	62.0	65.8	70.4	73.7	76.6	79.2	81.8	84.7	88.0	92.6	96.4
17.0–17.9 years	93	0.98	82.2	0.131	64.5	68.4	73.1	76.6	79.5	82.2	84.9	87.8	91.3	96.0	99.9
Girls (*N* = 833)															
12.0–12.9 years	160	0.50	72.9	0.137	57.4	60.7	64.7	67.8	70.4	72.9	75.4	78.2	81.6	86.3	90.3
13.0–13.9 years	211	0.48	74.2	0.136	58.6	61.8	66.0	69.0	71.7	74.2	76.8	79.6	83.0	87.7	91.8
14.0–14.9 years	144	0.37	74.3	0.133	59.1	62.3	66.3	69.2	71.8	74.3	76.8	79.6	82.9	87.7	91.8
15.0–15.9 years	116	0.61	75.6	0.132	59.9	63.2	67.4	70.4	73.1	75.6	78.1	80.9	84.2	88.8	92.7
16.0–16.9 years	130	0.99	76.9	0.129	60.6	64.2	68.6	71.7	74.4	76.9	79.4	82.1	85.3	89.6	93.3
17.0–17.9 years	72	1.01	78.5	0.127	62.1	65.7	70.1	73.3	76.0	78.5	81.0	83.7	86.9	91.3	94.9

**Table 4:** Smoothed percentile standards for body surface area (BSA) and waist-to-height ratio (WHtR) according to age and sex in Québec adolescents.

BSA (m^2^)					Percentiles										
Age	N	L	M	S	P_5_	P_10_	P_20_	P_30_	P_40_	P_50_	P_60_	P_70_	P_80_	P_90_	P_95_
Boys (*N* = 982)															
12.0–12.9 years	128	−0.60	1.48	0.150	1.18	1.23	1.31	1.37	1.43	1.48	1.54	1.60	1.69	1.82	1.93
13.0–13.9 years	223	0.07	1.57	0.140	1.24	1.31	1.39	1.46	1.52	1.57	1.63	1.69	1.77	1.88	1.97
14.0–14.9 years	170	−2.14	1.65	0.115	1.41	1.45	1.51	1.56	1.60	1.65	1.70	1.76	1.84	1.97	2.10
15.0–15.9 years	208	−1.17	1.72	0.115	1.45	1.50	1.57	1.62	1.67	1.72	1.77	1.83	1.91	2.02	2.13
16.0–16.9 years	161	−1.26	1.79	0.112	1.52	1.57	1.64	1.69	1.74	1.79	1.84	1.90	1.98	2.10	2.21
17.0–17.9 years	93	−2.75	1.82	0.119	1.56	1.60	1.67	1.72	1.77	1.82	1.88	1.95	2.05	2.22	2.41
Girls (*N* = 838)															
12.0–12.9 years	162	−1.60	1.50	0.142	1.23	1.28	1.34	1.40	1.45	1.50	1.56	1.62	1.71	1.86	2.01
13.0–13.9 years	212	−0.10	1.52	0.132	1.23	1.29	1.36	1.42	1.47	1.52	1.57	1.63	1.70	1.80	1.89
14.0–14.9 years	144	−1.99	1.59	0.131	1.33	1.38	1.44	1.49	1.54	1.59	1.65	1.71	1.80	1.95	2.11
15.0–15.9 years	117	−1.44	1.60	0.093	1.39	1.43	1.49	1.53	1.56	1.60	1.64	1.68	1.74	1.82	1.90
16.0–16.9 years	131	−0.80	1.65	0.108	1.40	1.45	1.51	1.56	1.61	1.65	1.70	1.75	1.81	1.91	2.00
17.0–17.9 years	71	−0.78	1.66	0.126	1.37	1.43	1.50	1.56	1.61	1.66	1.71	1.78	1.85	1.97	2.08
WHtR (ratio)					Percentiles										
Age	N	L	M	S	P_5_	P_10_	P_20_	P_30_	P_40_	P_50_	P_60_	P_70_	P_80_	P_90_	P_95_
Boys (*N* = 976)															
12.0–12.9 years	125	1.01	0.45	0.128	0.36	0.38	0.40	0.42	0.44	0.45	0.46	0.48	0.50	0.52	0.54
13.0–13.9 years	223	0.25	0.45	0.130	0.36	0.38	0.40	0.42	0.44	0.45	0.46	0.48	0.50	0.53	0.55
14.0–14.9 years	172	0.21	0.45	0.128	0.36	0.38	0.40	0.42	0.44	0.45	0.46	0.48	0.50	0.53	0.55
15.0–15.9 years	206	0.70	0.46	0.128	0.37	0.39	0.41	0.43	0.45	0.46	0.47	0.49	0.51	0.54	0.56
16.0–16.9 years	156	−0.60	0.47	0.128	0.39	0.40	0.42	0.44	0.46	0.47	0.49	0.50	0.53	0.56	0.59
17.0–17.9 years	93	1.59	0.48	0.146	0.35	0.38	0.42	0.44	0.46	0.48	0.50	0.52	0.54	0.57	0.59
Girls (*N* = 833)															
12.0–12.9 years	160	0.01	0.47	0.128	0.38	0.40	0.42	0.44	0.46	0.47	0.49	0.50	0.52	0.55	0.58
13.0–13.9 years	211	−0.31	0.47	0.146	0.38	0.40	0.42	0.44	0.46	0.47	0.49	0.50	0.52	0.56	0.58
14.0–14.9 years	144	0.71	0.48	0.128	0.37	0.39	0.42	0.44	0.46	0.48	0.50	0.52	0.54	0.57	0.60
15.0–15.9 years	116	1.01	0.46	0.104	0.36	0.38	0.41	0.43	0.45	0.46	0.47	0.49	0.51	0.54	0.56
16.0–16.9 years	130	−0.80	0.48	0.122	0.41	0.42	0.44	0.46	0.47	0.48	0.49	0.51	0.53	0.55	0.58
17.0–17.9 years	72	1.01	0.48	0.128	0.38	0.40	0.43	0.45	0.47	0.48	0.49	0.51	0.53	0.56	0.58

**Table 5:** Secular trend for waist circumference (WC) between the 1981 Canadian Fitness Survey (CFS) and 2017 current study in adolescents.

Age (Years)	N	CFS 1981 (cm)	N	Current Study 2017 (cm)	*p* Values	Cohen’s d Effect Size	∆ WC (cm)
Boys							
12	187	66.7 ± 7.1	125	73.2 ± 10.6	0.0001	0.75	6.5
13	218	67.5 ± 5.4	223	74.8 ± 10.3	0.0001	0.89	7.3
14	189	70.8 ± 6.5	170	76.4 ± 10.6	0.0001	0.65	5.6
15	191	73.7 ± 6.6	206	78.3 ± 10.3	0.0001	0.55	4.6
16	204	74.9 ± 6.3	157	80.2 ± 10.2	0.0001	0.64	5.3
17	187	76.7 ± 9.0	92	82.3 ± 10.1	0.0001	0.60	5.6
Mean							$\bar{x}$ = 5.8
Girls							
12	208	64.5 ± 7.9	161	73.5 ± 10.1	0.0001	1.01	9.0
13	184	67.0 ± 6.9	211	74.5 ± 10.1	0.0001	0.86	7.5
14	181	68.2 ± 7.3	143	75.7 ± 9.8	0.0001	0.88	7.5
15	200	70.8 ± 7.7	117	76.6 ± 10.1	0.0001	0.67	5.8
16	185	70.5 ± 7.9	128	77.7 ± 10.0	0.0001	0.82	7.2
17	195	71.5 ± 9.0	71	78.6 ± 10.0	0.0001	0.77	7.1
Mean							$\bar{x}$ = 7.4

*N* = number of participants; CFS = Canadian Fitness Survey; WC = waist circumference; *p* values = significant ≤ 0.05; Cohen’s d effect size: <0.2 = trivial; 0.2–0.5 = small; 0.5–0.8 = moderate; >0.8 = high; $\bar{x}$ = mean.

**Table 6:** Classification of individual health markers based on age and gender-specific risk zones.

	* BMI (kg·m^−2^)					
	Boys			Girls		
Age	Low-Risk	High-Risk	Very-High-Risk	Low-Risk	High-Risk	Very-High-Risk
12	≤21.5	21.6–26.4	≥26.5	≤22.0	22.1–27.2	≥27.3
13	≤22.2	22.3–27.2	≥27.3	≤22.9	23.0–28.1	≥28.2
14	≤22.9	23.0–27.9	≥28.0	≤23.6	23.7–28.8	≥28.9
15	≤23.5	23.6–28.5	≥28.6	≤24.1	24.2–29.2	≥29.3
16	≤24.1	24.1–29.1	≥29.2	≤24.5	24.6–29.5	≥29.6
17	≤24.6	24.7–29.6	≥29.7	≤24.8	24.9–29.7	≥29.8
	WC					
12	≤81.6	81.7–90.0	≥90.1	≤81.5	81.6–90.2	≥90.3
13	≤81.9	82.0–90.1	≥90.2	≤82.9	83.0–91.7	≥91.8
14	≤84.6	84.7–93.1	≥93.2	≤82.8	82.9–91.7	≥91.8
15	≤87.0	87.1–95.5	≥95.6	≤84.1	84.2–92.6	≥92.7
16	≤87.9	88.0–96.3	≥96.4	≤85.2	85.3–93.2	≥93.3
17	≤91.3	91.4–99.8	≥99.9	≤86.8	86.9–94.8	≥94.9
	WHtR (Ratio)					
12	≤0.49	0.50–0.53	≥0.54	≤0.51	0.52–0.57	≥0.58
13	≤0.49	0.50–0.54	≥0.55	≤0.51	0.52–0.57	≥0.58
14	≤0.49	0.50–0.54	≥0.55	≤0.53	0.54–0.59	≥0.60
15	≤0.50	0.51–0.55	≥0.56	≤0.50	0.51–0.55	≥0.56
16	≤0.52	0.53–0.58	≥0.59	≤0.52	0.53–0.57	≥0.58
17	≤0.53	0.54–0.58	≥0.59	≤0.52	0.53–0.57	≥0.58
	BSA (m^2^)					
12	≤1.68	1.69–1.92	≥1.93	≤1.70	1.71–2.00	≥2.01
13	≤1.76	1.77–1.96	≥1.97	≤1.69	1.70–1.89	≥1.90
14	≤1.84	1.85–2.09	≥2.10	≤1.79	1.80–2.10	≥2.11
15	≤1.91	1.92–2.12	≥2.13	≤1.73	1.74–1.90	≥1.91
16	≤1.97	1.98–2.20	≥2.21	≤1.80	1.81–2.00	≥2.01
17	≤2.04	2.05–2.40	≥2.41	≤1.84	1.85–2.07	≥2.08
	VO_2_max (ml·kg^−1^·min^−1^)					
12	≥44.6	44.5–36.6	≤36.5	≥41.4	41.3–34.7	≤34.6
13	≥43.7	43.6–36.3	≤36.2	≥39.4	39.3–32.2	≤32.1
14	≥44.3	44.2–34.1	≤34.0	≥37.2	37.1–30.7	≤30.6
15	≥42.9	42.8–32.2	≤32.1	≥38.2	38.1–29.7	≤29.6
16	≥42.7	42.6–31.5	≤31.4	≥35.9	35.8–26.8	≤26.7
17	≥42.0	41.9–29.5	≤29.4	≥34. 7	34.6–25.3	≤25.2
	Stages (number)					
12	≥5.1	5.0–2.1	≤2.0	≥3.7	3.6–1.1	≤1.0
13	≥5.3	5.2–2.4	≤2.3	≥3.7	3.6–1.0	≤0.9
14	≥6.1	6.0–2.4	≤2.3	≥3.4	3.3–1.0	≤0.9
15	≥6.1	6.0–2.4	≤2.3	≥4.5	4.4–1.5	≤1.4
16	≥6.6	6.5–2.8	≤2.7	≥4.3	4.2–1.2	≤1.1
17	≥6.6	6.5–2.8	≤2.7	≥4.1	4.0–1.3	≤1.2

Age = years; * based on WHO cut off points for age and sex.

**Table 7:** Paired-*t*-test comparison of measured and predicted percentile values for composite scores.

	N	Mean ± (SD)	CI	SEM	*p* Value	Cohen’s d	R^2^
Percentile Based on Four Markers ∑(BMI, WC, VO_2_peak, Stages)/4							
Measured composite score (Percentile)	1791	49.8 ± 23.0	48.7–50.8	0.542	0.783	0.000	0.891
Predicted composite score (Percentile)	1791	49.8 ± 21.8	48.8–50.8	0.512			
Percentile based on three markers ∑(BMI, WC, VO_2_peak)/3							
Measured composite score (Percentile)	1798	49.8 ± 23.3	48.7–50.9	0.550	0.016	0.018	0.881
Predicted composite score (Percentile)	1798	49.4 ± 21.9	48.3–50.4	0.515			

N = number of participants; SD = standard deviation; CI = confidence interval; SEM = standard error of the mean; *p* = significant at *p* ≤ 0.05; Cohen’s d = effect size; R^2^ = coefficient of determination.

**Table 8:** Comparison of two models for early detection of cardiometabolic risk in adolescents.

	R^2^	RMSE	MAE	SEE	Mean VIF
Equation (1) Composite score estimation with the average percentile of four markers					
Composite score = −15.54 + (WC × 0.91) + (BMI × 1.45) + (Stage × −4.62) + (VO_2_peak × −0.298)	0.891	7.5	6.0	7.5	3.3 Range: 2.5–4.1
Equation (2) Composite score estimation with the average percentile of three markers (without stage)					
Composite score = −23.26 + (WC × 1.00) + (BMI × 2.09) + (VO_2_peak × −1.20)	0.881	8.1	6.5	8.1	2.0 Range: 1.1–2.5

R^2^ = coefficient of determination; RMSE = root mean square error; MAE = mean absolute error; SEE = standard error of estimate; VIF = variance inflation factor.

**Table 9:** Percentage of adolescents who present a health risk according to anthropometric and CRF markers according to age and sex.

Age	BMI	WC	WHtR	BSA	VO_2_peak	Stages	Composite Score
Boys							
12	32.8%	22.4%	32.0%	15.6%	49.2%	54.7%	34.5%
13	30.5%	25.9%	27.8%	15.7%	45.8%	48.4%	32.4%
14	23.3%	21.1%	22.8%	14.0%	45.3%	54.1%	30.1%
15	24.5%	21.4%	22.3%	16.7%	47.0%	52.1%	30.7%
16	24.9%	23.6%	13.4%	18.6%	50.3%	52.8%	30.6%
17	28.3%	21.5%	16.1%	16.1%	57.3%	52.1%	31.9%
Mean	27.4%	22.7%	22.4%	16.1%	49.2%	52.4%	31.7%
Girls							
12	29.6%	28.8%	27.5%	13.0%	51.8%	51.2%	33.7%
13	24.9%	21.3%	21.3%	17.9%	53.0%	57.0%	32.6%
14	34.7%	29.2%	21.5%	19.4%	55.2%	53.1%	35.5%
15	22.9%	21.6%	23.3%	21.2%	55.7%	53.2%	33.0%
16	22.1%	20.8%	18.5%	16.0%	48.9%	52.6%	29.8%
17	22.5%	20.8%	26.4%	16.9%	65.8%	56.9%	34.9%
Mean	26.1%	23.8%	23.1%	17.4%	55.1%	54.0%	33.3%

Age = years; BMI = body mass index; WC = waist circumference; WHtR = waist-to-height ratio; BSA = body surface area; VO_2_peak = estimated by the 20 m shuttle run test; Stages = number of 1 min stages completed during the 20 m shuttle run test: Composite score = mean of % all markers.

## Data Availability

The original contributions presented in the study are included in the article/Supplementary Material; further inquiries can be directed to the corresponding author/s.

## Supplementary Materials

The following supporting information can be downloaded at: https://www.mdpi.com/article/10.3390/ijerph21040408/s1, File S1: Excel spreadsheet to assess the risk associated for each specific marker and the overall composite risk.

## References

[1] Cardel M.I., Atkinson M.A., Taveras E.M., Holm J.C., Kelly A.S. (2020). Obesity treatment among adolescents: A review of current evidence and future directions. JAMA Pediatr..

[2] Bailey D.P., Boddy L.M., Savory L.A., Denton S.J., Kerr C.J. (2012). Associations between cardiorespiratory fitness, physical activity and clustered cardiometabolic risk in children and adolescents: The HAPPY study. Eur. J. Pediatr..

[3] Bravo J., Raimundo A.M., Santos D.A., Timón R., Sardinha L.B. (2017). Abdominal obesity in adolescents: Development of age-specific waist circumference cut-offs linked to adult IDF criteria. Am. J. Hum. Biol..

[4] Cornier M.-A., Després J.-P., Davis N., Grossniklaus D.A., Klein S., Lamarche B., Lopez-Jimenez F., Rao G., St-Onge M.-P., Towfighi A. (2011). Assessing adiposity: A scientific statement from the American Heart Association. Circulation.

[5] Juonala M., Magnussen C.G., Berenson G.S., Venn A., Burns T.L., Sabin M.A., Srinivasan S.R., Daniels S.R., Davis P.H., Chen W. (2011). Childhood adiposity, adult adiposity, and cardiovascular risk factors. N. Engl. J. Med..

[6] Becque M.D., Hattori K., Katch V.L., Rocchini A.P. (1992). Fat patterning of adolescents: Allometry of fatfolds. Am. J. Hum. Biol..

[7] Figueroa-Colon R., Mayo M.S., Treuth M.S., Aldridge R., Hunter G., Berland L., Goran M., Weinsier R. (1998). Variability of abdominal adipose tissue measurements using computed tomography in prepubertal girls. Int. J. Obes. Relat. Metab. Disord..

[8] Gutin B., Barbeau P., Owens S., Lemmon C.R., Bauman M., Allison J., Kang H.-S., Litaker M.S. (2002). Effects of exercise intensity on cardiovascular fitness, total body composition, and visceral adiposity of obese adolescents. Am. J. Clin. Nutr..

[9] Watts K., Jones T.W., Davis E.A., Green D. (2005). Exercise training in obese children and adolescents: Current concepts. Sports Med..

[10] Taylor R.W., Jones I.E., Williams S.M., Goulding A. (2000). Evaluation of waist circumference, waist-to-hip ratio, and the conicity index as screening tools for high trunk fat mass, as measured by dual-energy X-ray absorptiometry, in children aged 3–19 y. Am. J. Clin. Nutr..

[11] Cameron N. (2007). Body mass index cut offs to define thinness in children and adolescents. BMJ.

[12] Chung S. (2015). Body mass index and body composition scaling to height in children and adolescent. Ann. Pediatr. Endocrinol. Metab..

[13] Karchynskaya V., Kopcakova J., Klein D., Gába A., Madarasova-Geckova A., van Dijk J.P., de Winter A.F., Reijneveld S.A. (2020). Is BMI a valid indicator of overweight and obesity for adolescents?. Int. J. Environ. Res. Public Health.

[14] Cole T.J., Bellizzi M.C., Flegal K.M., Dietz W.H. (2000). Establishing a standard definition for child overweight and obesity worldwide: International survey. BMJ.

[15] Dundar I., Akinci A. (2023). Prevalence and Predictive Clinical Characteristics of Metabolically Healthy Obesity in Obese Children and Adolescents. Cureus.

[16] Franks P.W., Hanson R.L., Knowler W.C., Sievers M.L., Bennett P.H., Looker H.C. (2010). Childhood obesity, other cardiovascular risk factors, and premature death. N. Engl. J. Med..

[17] Skinner A.C., Perrin E.M., Moss L.A., Skelton J.A. (2015). Cardiometabolic risks and severity of obesity in children and young adults. N. Engl. J. Med..

[18] Janssen I., Shields M., Craig C.L., Tremblay M.S. (2011). Prevalence and secular changes in abdominal obesity in Canadian adolescents and adults, 1981 to 2007–2009. Obes. Rev..

[19] Xi B., Zong X., Kelishadi R., Litwin M., Hong Y.M., Poh B.K., Steffen L.M., Galcheva S.V., Herter-Aeberli I., Nawarycz T. (2020). International Waist Circumference Percentile Cutoffs for Central Obesity in Children and Adolescents Aged 6 to 18 Years. J. Clin. Endocrinol. Metab..

[20] Kiess W., Galler A., Reich A., Müller G., Kapellen T., Deutscher J., Raile K., Kratzsch J. (2001). Clinical aspects of obesity in childhood and adolescence. Obes. Rev..

[21] Lee X., Gao Y., Zhang Y., Feng Y., Gao L., Wang A., Jiang Y., Huang H. (2022). Comparison of 10 obesity-related indices for predicting hypertension based on ROC analysis in Chinese adults. Front. Public Health.

[22] Krakauer N.Y., Krakauer J.C. (2012). A new body shape index predicts mortality hazard independently of body mass index. PLoS ONE.

[23] Alberti K.G., Zimmet P., Shaw J. (2006). Metabolic syndrome–a new worldwide definition. A Consensus Statement from the International Diabetes Federation. Diabet. Med..

[24] Ashwell M., Gibson S. (2016). Waist-to-height ratio as an indicator of ‘early health risk’: Simpler and more predictive than using a ‘matrix’ based on BMI and waist circumference. BMJ Open..

[25] Brambilla P., Bedogni G., Heo M., Pietrobelli A. (2013). Waist circumference-to-height ratio predicts adiposity better than body mass index in children and adolescents. Int. J. Obes..

[26] McCarthy H.D., Ashwell M. (2006). A study of central fatness using waist-to-height ratios in UK children and adolescents over two decades supports the simple message--’keep your waist circumference to less than half your height’. Int. J. Obes..

[27] Lee C.M., Huxley R.R., Wildman R.P., Woodward M. (2008). Indices of abdominal obesity are better discriminators of cardiovascular risk factors than BMI: A meta-analysis. J. Clin. Epidemiol..

[28] Savva S., Tornaritis M., Savva M., Kourides Y., Panagi A., Silikiotou N., Georgiou C., Kafatos A. (2000). Waist circumference and waist-to-height ratio are better predictors of cardiovascular disease risk factors in children than body mass index. Int. J. Obes. Relat. Metab. Disord..

[29] Sharma A.K., Metzger D.L., Daymont C., Hadjiyannakis S., Rodd C.J. (2015). LMS tables for waist-circumference and waist-height ratio Z-scores in children aged 5–19 y in NHANES III: Association with cardio-metabolic risks. Pediatr. Res..

[30] Ashby-Thompson M., Ji Y., Wang J., Yu W., Thornton J.C., Wolper C., Weil R., Chambers E.C., Laferrère B., Pi-Sunyer F.X. (2020). High-resolution three-dimensional photonic scan-derived equations improve body surface area prediction in diverse populations. Obesity.

[31] Cléro E., Leux C., Brindel P., Truong T., Anger A., Teinturier C., Diallo I., Doyon F., Guénel P., de Vathaire F. (2010). Pooled analysis of two case-control studies in New Caledonia and French Polynesia of body mass index and differentiated thyroid cancer: The importance of body surface area. Thyroid.

[32] Sardinha L.B., Silva A.M., Minderico C.S., Teixeira P.J. (2006). Effect of body surface area calculations on body fat estimates in non-obese and obese subjects. Physiol. Meas..

[33] Si S., Tewara M.A., Ji X., Wang Y., Liu Y., Dai X., Wang Z., Xue F. (2020). Body surface area, height, and body fat percentage as more sensitive risk factors of cancer and cardiovascular disease. Cancer Med..

[34] Yu X.H., Cao R.R., Yang Y.Q., Deng F.Y., Bo L., Lei S.F. (2023). Body surface area is a potential obesity index: Its genetic determination and its causality for later-life diseases. Obesity.

[35] Buchan D.S., Young J.D., Boddy L.M., Malina R.M., Baker J.S. (2013). Fitness and adiposity are independently associated with cardiometabolic risk in youth. BioMed Res. Int..

[36] Raghuveer G., Hartz J., Lubans D.R., Takken T., Wiltz J.L., Mietus-Snyder M., Al-Mutairi M.E. (2020). Cardiorespiratory Fitness in Youth: An Important Marker of Health: A Scientific Statement from the American Heart Association. Circulation.

[37] Ruiz J.R., Ortega F.B., Rizzo N.S., Villa I., Hurtig-Wennlöf A., Oja L., Sjöström M. (2007). High cardiovascular fitness is associated with low metabolic risk score in children: The European Youth Heart Study. Pediatr. Res..

[38] Ruiz J.R., Cavero-Redondo I., Ortega F.B., Welk G.J., Andersen L.B., Martinez-Vizcaino V. (2016). Cardiorespiratory fitness cut points to avoid cardiovascular disease risk in children and adolescents; what level of fitness should raise a red flag? A systematic review and meta-analysis. Br. J. Sports Med..

[39] Ross R., Blair S.N., Arena R., Church T.S., Despres J.P., Franklin B.A., Haskell W.L., Kaminsky L.A., Levine B.D., Lavie C.J. (2016). Importance of assessing cardiorespiratory fitness in clinical practice: A case for fitness as a clinical vital sign: A scientific statement from the American Heart Association. Circulation.

[40] Léger L.A., Mercier D., Gadoury C., Lambert J. (1988). The multistage 20 metre shuttle run test for aerobic fitness. J. Sports Sci..

[41] Tomkinson G.R., Lang J.J., Tremblay M.S., Dale M., LeBlanc A.G., Belanger K., Ortega F.B., Léger L. (2017). International normative 20 m shuttle run values from 1,142,026 children and youth representing 50 countries. Br. J. Sports Med..

[42] Olds T., Tomkinson G., Léger L., Cazorla G. (2006). Worldwide variation in the performance of children and adolescents: An analysis of 109 studies of the 20-m shuttle run test in 37 countries. J. Sports Sci..

[43] Leone M., Levesque P., Bourget-Gaudreault S., Lemoyne J., Kalinova E., Comtois A.S., Bui H.T., Léger L., Frémont P., Allisse M. (2023). Secular trends of cardiorespiratory fitness in children and adolescents over a 35-year period: Chronicle of a predicted foretold. Front. Public Health.

[44] Kalton G., Anderson D.J. (1986). Introduction to Survey Sampling.

[45] Lohman T.G., Roche A.F., Martorell R. (1988). Anthropometric Standardization Reference Manual.

[46] Canadian Society for Exercise Physiology (CSEP) (2003). The Canadian Physical Activity, Fitness and Lifestyle Approach.

[47] Mosteller R.D. (1987). Simplified calculation of body-surface area. N. Engl. J. Med..

[48] World Health Organization (2006). WHO Child Growth Standards: Length/Height-for-Age, Weight-for-Age, Weight-for-Length, Weight-for-Height and Body Mass Index-for-Age: Methods and Development.

[49] Hoaglin D.C., Iglewicz B. (1987). Fine tuning some resistant rules for outlier labeling. J. Am. Stat. Assoc..

[50] Cole T.J., Green P.J. (1992). Smoothing reference centile curves: The LMS method and penalized likelihood. Stat. Med..

[51] Leone M., Bui H.T., Kalinova E., Bourget-Gaudreault S., Levesque P., Lemoyne J., Gagnon D., Larivière G., Léger L., Allisse M. (2023). Updating normative cross-sectional values and secular trends in body mass, body height and body mass index among Québec children and adolescents. Can. J. Public Health.

[52] Kuhle S., Maguire B., Ata N., Hamilton D. (2015). Percentile Curves for Anthropometric Measures for Canadian Children and Youth. PLoS ONE.

[53] Johansson L., Putri R.R., Danielsson P., Hagströmer M., Marcus C. (2023). Associations between cardiorespiratory fitness and cardiometabolic risk factors in children and adolescents with obesity. Sci. Rep..

[54] Patry-Parisien J., Shields M., Bryan S. (2012). Comparison of waist circumference using the World Health Organization and National Institutes of Health protocols. Health Rep..

[55] Katzmarzyk P.T. (2004). Waist circumference percentiles for Canadian youth 11–18 y of age. Eur. J. Clin. Nutr..

